# Breast cancer bone metastasis and bone metastatic cells retain NKG2DLs intracellularly: could this be a strategy to evade immune recognition?

**DOI:** 10.3389/fcell.2026.1717607

**Published:** 2026-01-27

**Authors:** Marta Gomarasca, Paola Maroni, Chiara Verdelli, Laura Gerosa, Martina Faraldi, Alessandro Luzzati, Luca Cannavò, Giuseppe Banfi, Giovanni Lombardi

**Affiliations:** 1 Laboratory of Experimental Biochemistry & Advanced Diagnostics, IRCCS Ospedale Galeazzi-Sant’Ambrogio, Milano, Italy; 2 Oncological and Reconstructive Surgery Unit, IRCCS Ospedale Galeazzi-Sant’Ambrogio, Milano, Italy; 3 Orthopedics and Traumatology Unit, Ospedale di Esine ASST Valcamonica, Esine, Italy; 4 Vita-Salute San Raffaele University, Milano, Italy; 5 Department of Athletics, Strength and Conditioning, Poznań University of Physical Education, Poznań, Poland

**Keywords:** bone metastasis, breast cancer, breast cancer immune evasion, NK, NKG2D, NKG2DLs

## Abstract

Bone metastases dramatically worsen breast cancer (BC) prognosis reducing overall survival. Natural killer (NK) cells recognize and eliminate malignant cells through the interaction with NKG2D receptor ligands (NKG2DLs) on cancer cells. Tumors often evade NK surveillance by downregulating the NKG2DLs expression and avoid recognition, but whether this occurs in bone metastases remains unclear. This study investigates mechanisms of NKG2DLs downregulation in primary BC and bone metastases (BoMet). Expression and localization of the NKG2D/NKG2DL axis components were investigated in BC tissues (with and without metastases), paired bone metastatic ductal carcinoma (bmDC), BoMet, and in BC cells lines of varying invasiveness. In bmDC and BoMet, major histocompatibility complex class I chain-related protein A and B (MICA/B) and UL16-binding protein 2 (ULBP2) localized in perinuclear area, contrasting with predominantly cytosolic distribution in non-metastatic BC. Similarly, invasive MDA-MB-231 and MDA-BoM-1833 showed NKG2DLs perinuclear localization and co-localization with the Golgi apparatus, while less invasive MCF7 showed a prominent cytosolic distribution. Accumulation of NKG2DLs in membrane and cytoskeletal fractions further supports this pattern. Additionally, when N-glycosilation is impaired, NKG2DLs fail to reach the cell surface in metastatic cell lines, while are still transported through the Golgi apparatus and delivered to the plasma membrane, resulting in increased surface expression irrespective of correct glycosylation. Our findings suggest that invasive and bone-metastatic breast cancer cells are more dependent on correct glycosylation and intracellular trafficking for NKG2DL surface expression than non-metastatic breast cancer cells. This difference may have important implications for potential immune evasion mechanisms and for the development of therapeutic strategies targeting bone metastases in breast cancer.

## 1 Introduction

Breast cancer (BC) is the prevalent tumor among women, with the bone being the most frequent site of metastasis. The development of metastatic disease significantly worsens prognosis and is responsible for cancer-related deaths due to its impact on reducing overall survival rates (Chaffe et al., 2011).

Immune cells play a critical role in the tumor microenvironment, where they contribute to immune surveillance and in this context, natural killer (NK) cells are particularly important for their ability to recognize and eliminate malignant cells. NK cells, an important compartment of the innate immunity, detect stress-induced ligands, the NKG2D receptor ligands (NKG2DLs), which are deregulated on the surface of transformed cells. Such ligands, major histocompatibility complex class I chain-related protein A and B (MICA/B) and UL16-binding protein 1–6 (ULBP1-6), are recognized by NKG2D, a key activation receptor expressed on NK cells, cytotoxic T lymphocytes and other T-cell subpopulations (Ogasawara and Lanier, 2005). NK cells play a vital role in immune surveillance by identifying and eliminating cells that lack “self” markers (like MHC class I) or express stress-induced ligands, such as NKG2DLs. When these ligands are expressed on the surface of tumor cells, they engage the activating receptor NKG2D on NK cells, triggering cytotoxic responses mediated by the release of cytotoxic factors such as perforin and granzyme, and trigger apoptosis in tumor cells (Lopez-Soto et al., 2017a).

While NKG2DLs are absent or low expressed in most normal cells (Cerboni et al., 2014), their expression is induced upon stress and malignant transformation. However, cancer cells often develop strategies to escape this immune surveillance, thereby promoting disease progression and metastasis (Schmiedel and Mandelboim, 2018). Indeed, neoplastic cells employ a specialized immune evasion strategy characterized by the proteolytic shedding of surface-expressed NKG2DLs via tumor-derived metalloproteases. This enzymatic cleavage generates soluble NKG2DLs, which function as immunomodulatory decoys, impairing NKG2D receptor engagement on NK cells and thereby attenuating NK cell–mediated cytotoxicity, ultimately facilitating tumor immune escape (Lopez-Soto et al., 2017b).

Conversely, other tumors upregulate NKG2DL expression, which paradoxically leads to the downregulation of the NKG2D receptor on NK cells due to sustained ligand engagement, resulting in diminished NK cell responsiveness (Wensveen et al., 2018; Groh et al., 2002). This adaptive mechanism serves to mitigate NK cell hyper-activation in the presence of persistent NKG2DL expression. Targeted modulation of the NKG2D–NKG2DL axis represents a promising immunotherapeutic approach for the treatment of a broad spectrum of malignancies (Long et al., 2013). Another proposed mechanism by which cancer cells evade NK cell-mediated killing is the intracellular retention and reduced or absent surface expression of NKG2DLs. This immune evasion strategy has been documented in several melanoma cell lines (Fuertes et al., 2008). The intracellular trapping may result from altered post-translational processing, defective vesicular transport, or active degradation pathways. Understanding the mechanisms that regulate NKG2DL surface expression is critical, as it opens therapeutic avenues. For example, drugs that enhance NKG2DL expression or prevent their degradation could sensitize tumors to NK cell-mediated killing, improving the efficacy of immunotherapies.

In the present study, we evaluated the expression profile and localization of NKG2DLs in human biopsies from two types of invasive breast cancer, as well as in bone metastatic samples paired with breast cancer. Using a different technical approach, we investigated *in vitro* whether aggressive breast cancer cells and metastatic cells have an active mechanism that allows them to evade NK immune surveillance by downregulating the expression of NKG2DLs.

## 2 Materials and methods

### 2.1 Human breast cancer and bone metastasis from breast cancer tissue samples

Bone metastases (BoMet) (n = 10) were collected during surgical interventions at IRCCS Ospedale Galeazzi-Sant’Ambrogio, Milano, Italy, while the paired bone metastatic ductal carcinomas (bmDC) were provided by the hospital where the surgery was performed (n = 7). This is an observational, cross-sectional study on basic research and it was approved by the ethical committee (Ospedale San Raffaele, Milan, Italy; Ref. No. 176/INT/2019) and registered at clinicaltrials.gov (NCT04167605). All subjects gave their written informed consent to participate in the study in accordance with the Declaration of Helsinki. All samples, collected from January 2020 to September 2023, were anonymized, and no information or images that could lead to the identification of a study participant might ever occur.

Tissue microarray (TMA) with adjacent normal tissue (ANT) (n = 20), triple negative breast cancer (TNBC) (n = 33), and breast cancer Luminal A type (n = 47) were obtained from TissueArray.com (BC08032, BR1202, and BR1507).

### 2.2 Immunohistochemistry

BoMet specimens were fixed in 10% neutral buffered formalin, decalcified in Mielodec (04-230827/B, Bio-Optica), embedded in paraffin and serial sections of 4 μm were obtained under microtome. One section for each specimen of bone metastases and carcinomas was stained with hematoxylin and eosin (H&E), and two serial sections for each specimen were processed for specific immunostaining. After antigen retrieval (95 °C for 20 min, at pH 6, in antigen-unmasking solution, H-3300, Vector Laboratories), sections were treated for 10 min with 0.1% (v/v) H_2_O_2_ and blocked with normal serum. Each specimen was assayed with anti-MICA/B [F-6] (1:50) (sc-137242, Santa Cruz Biotechnology), and anti-ULBP2 (1:300) (MA5-29636, Invitrogen, ThermoFisher Scientific) antibodies. Immunostaining was performed with a streptavidin-biotin system (PK-6102 VECTASTAIN® Elite® ABC Kit, Peroxidase (Mouse IgG) or PK-6101VECTASTAIN® Elite® ABC-HRP Kit, Peroxidase (Rabbit IgG), Vector Laboratories) and diaminobenzidine substrate (RE7230-CE, NovoLink DAB, Leica Biosystems) and counterstained with Mayer’s hematoxylin (109249, Merck) (Maroni et al., 2015). Negative controls were subjected to the same staining procedure in which the primary antibody was omitted. The chosen antibodies were validated for immunohistochemistry analysis (IHC) by the manufacturers and were tested in pilot experiments by titration.

Specimens were analyzed under a microscope (CKX41 Olympus Corp., Tokio, Japan). Immunohistochemical staining was evaluated considering the percentage of stained cells and the immunostaining intensity. Specimens with <10% of positive cells were considered negative. A semi-quantitative scale was applied: no staining (0); low staining (1); moderate staining (2), and strong staining (3).

### 2.3 Cell lines

The following cell lines were used: MDA-MD-231 (MDA-231) (HTB-26, ATCC), triple negative breast cancer (TNBC) invasive cells metastatic to the bones, brain, and lungs; MDA-BoM-1833 (1833), TNBC cells derived from MDA-MB-231 for its bone tropism (Kang et al., 2003) (purchased from Dr. Masseguè, Memorial-Sloan Kettering Cancer Center, United States); MCF7 (ER/PR^+^, HER2^-^ non-metastatic breast cancer cell line) (HTB-22, ATCC); MCF10A, normal human mammary cells (CRL-10317, ATCC). MDA-231 and 1833 were cultured in Dulbecco’s Modified Eagle Medium (DMEM, 10938-025, Gibco) supplemented with 10% HyClone™ foetal bovine serum (FBS, SV30160.03, GeHealthcare), 2 mM L-glutamine, 1 × 10^5^ U/L penicillin, 0.1 mg/L streptomycin (PSG, 10378-016, Gibco). MCF7 were cultured in RPMI-164 (R0883, Merck Millipore) supplemented with 10% FBS, 2 mM L-glutamine, 1 × 10^5^ U/L penicillin, 0.1 mg/L streptomycin, and 25 mM Hepes (15630-122, Gibco). MCF10A were cultured in DMEM/Ham’s F-12 (21331-020, Gibco) supplemented with 100 ng/mL cholera toxin (C8052, Merck Millipore), 20 ng/mL human epidermal growth factor (hEGF, SRP3027, Merck Millipore), 0.01 mg/mL insulin (I9278, Merck Millipore), 500 ng/mL hydrocortisone (H0888, Merck Millipore), and 5% heat-inactivated horse serum (H1270, Merck Millipore) (Qu et al., 2015).

Cells were grown in humidified incubators at 37 °C and 5% CO_2_. The medium was changed every second day, and the cells were passaged when they reached 80%–90% confluence. All cell lines were tested for *mycoplasma* by PCR and resulted negative.

For tunicamycin treatments, cells were starved in medium containing 1% FBS the day before and then treated with 1500 U tunicamycin (T7765, Merck Millipore) for 24 h, followed by cell lysis and SDS-PAGE analysis or flow cytometry analysis. For monensin treatments, cells were incubated with 2 µM monensin (554724, BD GolgiStop™ Protein Transport Inhibitor, BD Bioscience) for 3 h, before proceeding with immunofluorescence staining.

### 2.4 Immunofluorescence

For intracellular and co-localization staining, cells were fixed with 4% paraformaldehyde (PFA, J61899, ThermoFisher Scientific) for 10 min, at room temperature. Cells were permeabilized with 0.2% Triton X-100 (T9284, Merck Millipore) for 5 min, at room temperature, and blocked with blocking solution (3% BSA in PBS solution) for 1 h, at room temperature. Cells were then incubated with primary (overnight, at 4 °C) and secondary (45 min, at room temperature) antibodies diluted in antibody solution (blocking solution diluted 1:10 in PBS buffer). The following primary antibodies were used: MICA/B (1:50, sc-137242, Santa Cruz Biotechnology), ULBP2 (1:100, MA5-29636, Invitrogen), β-tubulin (1:500, T4026, Merck Millipore), β-tubulin (1:100, 2128, Cell Signaling Technology), calnexin (1:100, MA3-027, Invitrogen), calnexin (1:50, 2679, Cell Signaling Technology), GM130 (1:100, ab169276, AbCam), GM130 (1:3200, 1248, Cell Signaling Technology). Secondary antibodies conjugated to the following dies were used: Alexa Fluor 488 (1:1000, ab150105 or ab150073, Invitrogen); Alexa Fluor 568 (1:1000, ab175473 or ab175471, Invitrogen); Alexa Fluor 647 (1:1000, ab150115 or ab150079, Invitrogen). Nuclei were counterstained with 4′,6-diamidino-2-phenylindole (DAPI) 5 mg/μL (D9542, Merck Millipore) for 5 min, at room temperature.

For surface staining of the NKG2DLs, cells were fixed with 4% PFA for 10 min, at room temperature, blocked in 5% BSA (11943.03, Serva) diluted in PBS (BSA/PBS) for 45 min, at room temperature, and incubated with primary antibodies diluted in 0.5% BSA/PBS for 1.5 h, at room temperature. Then cells were incubated with secondary antibody diluted in 0.5% BSA/PBS for 1 h, at room temperature, and nuclei were counterstained with DAPI.

Images were acquired using a Leica SP8 laser scanning microscope (Leica Microsystems, Wetzlar, Germany) with oil immersion ×100 objectives. Confocal images were analyzed with Fiji software (Schindelin et al., 2012).

The co-localization between NKG2DLs and GM130 (marker for Golgi apparatus) or calnexin (marker for Endoplasmic Reticulum, ER) was quantified using the JACoP co-localization algorithm (Bolte and Cordelieres, 2006). The background was subtracted in each channel. Threshold was determined as 3*SD of the mean total intensity of fluorophore. Manders’ overlap coefficients were considered for the quantification of co-localization (Manders et al., 1993). In particular, it was considered the Manders’ overlap coefficient of red channel (organelles) overlapping with green channel (NKG2DLs). Finally, the Manders’ coefficient was normalized on the mean total intensity of the NKG2DL fluorophore to minimize the variability due to intensity, differences in cell line expression, and microscope configuration. Analysis was performed on six different fields for each condition across two independent experiments.

### 2.5 Flow cytometry

Flow cytometry analysis was performed to measure the amount for NKG2DL expressed either on the cell surface or intracellular. First, cells were trypsinized and fixed with 4% PFA diluted in FACS buffer (2% FBS in PBS) for 15 min, at room temperature. For surface staining cells were then blocked in FACS buffer for 45 min at room temperature and stained with MICA/B-APC (1:50, FAB13001A-100, Bio-Techne) and ULBP2-PE (1:50, FAB1298P, Bio-Techne), or ULBP2-APC (1:50, FAB1298A, Bio-Techne) antibodies diluted in FACS buffer for 45 min, at 4 °C. As control, cells were incubated with the isotype control of each antibody: IgG2A-APC (1:50, IC003A, Bio-Techne) and IgG2A-PE (1:50, IC003P, Bio-Techne), respectively.

For intracellular staining, after fixing, cells were permeabilized with 0.2% Tween-20 (P1379, Merck Millipore) diluted in PBS for 15 min, at 37 °C, blocked in FACS buffer for 45 min, at room temperature, and incubated with MICA/B-APC and ULBP2-PE antibodies, or isotype controls diluted in 0.1% Tween-20 (diluted in FACS buffer) for 45 min, at 4 °C.

Cells were analyzed with CytoFLEX cytometer (Beckman Coulter Inc., Brea, CA, United States) and data were analyzed with CytExpert software (Beckman Coulter Inc.). Data are expressed as ratio of mean fluorescent intensity of each protein to its isotype control.

### 2.6 SDS-PAGE and Western blot

Cells were lysed using RIPA lysis buffer (20-188, ThermoFisher Scientific) supplemented with protease and phosphatase inhibitors to obtain total protein extractions. Subcellular fractions (cytoplasmic, membrane, nuclear and cytoskeletal protein fractions) were obtained using the Subcellular Protein Fractionation Kit for Cultured Cells (78840, ThermoFisher Scientific). Total cell and subcellular fraction extracts were sonicated for three cycles of 11 s, at 40% amplitude, using the Vibra Cell ultrasonic processor (Sonics and Materials Inc.) to disrupt MICA/B high molecular weight aggregates.

Protein concentration was determined by the bicinchoninic acid (BCA) method with the Pierce BCA Protein Assay Kit (23225, ThermoFisher Scientific). Ten μg of cellular lysates were resolved on 10% gels by SDS-PAGE and transferred onto an Immobilon-P PVDF membrane (IPVH00005, Merck Millipore). As molecular weight reference, the Precision Plus Protein Dual Color standards (1610374, Bio-Rad Laboratories) was used. For immunoblotting the following primary antibodies were used: MICA/B (F-6) (1:500, sc-137242, Santa Cruz Biotechnology), ULBP2 (1:1000, ab275023, AbCam), GM130 (1:10000, 12480, Cell Signaling Technology). β-Actin (1:2000, TA811000, Origene), was used as loading control for total cell lysates and cytoplasmic fraction, while TATA-binding protein (TBP, 1:1000, ab51841, AbCam), and Na^+^/K^+^-ATPase (1:20000, ab76020, AbCam) were used as loading controls for nuclear and membrane fractions, respectively. Appropriate secondary antibodies, conjugated to horseradish peroxidase (1:10000, 115-035-003 or 111-035-003, Jackson ImmunoResearch Laboratories Inc.), were used and specific bands were detected with ChemiDoc Imaging System (Bio-Rad Laboratories). Acquired images were analyzed through Image Lab software (Bio-Rad Laboratories) and protein expression levels were normalized using the appropriate housekeeping as reference.

### 2.7 EndoH and PNGase F digestions

Twenty or 50 μg of total protein from breast cancer cell lysates were digested with either 1500 U of EndoH (V4871, Promega) for 4 h or 20 U of PNGase F (V4831, Promega) for 2 h, according to the manufacturer’s instructions. Following enzymatic digestion, MICA/B and ULBP2 expression was analyzed by SDS–PAGE and Western blotting, as described in the previous section.

### 2.8 Gene expression

Total RNA was extracted from cell cultures using TRIzol reagent (15596018, Invitrogen) following manufacturer’s instructions. RNA concentration was determined using NanoDrop spectrophotometer and DNA contamination was removed by DNaseI (18068015, Invitrogen) treatment. Reverse transcription reaction of 1 μg total DNA-free RNA was carried out using the iScript cDNA Synthesis Kit (1708891, Bio-Rad Laboratories) and RT-qPCR was performed on StepOne Plus System (Applied Biosystem, ThermoFisher Scientific) using the following Taqman gene expression assays: MICA (Hs00741286_m1), MICB (Hs00792952_m1), and ULBP2 (Hs00607609_m1). The reference genes ACTB (beta-actin, Hs99999903_m1) and GAPDH (Hs99999905_m1) were used to normalize expression data and relative gene expression was obtained using the 2^−ΔΔCT^ method. All the reagents and instruments were from ThermoFisher Scientific.

### 2.9 Statistical analysis

Statistical analysis was performed using GraphPad Prism version 8.3.0. Results from IHC quantitative analysis were analyzed by unpaired Student’s t-test. Results from Western blot quantification, real-time PCR, flow cytometry and co-localization analysis were analyzed using ordinary one-way ANOVA with Tukey’s multiple comparison and Kruskall-Wallis test with Dunn’s multiple comparison. Results from glycosylation inhibitors experiments were analyzed using two-way ANOVA followed by Sidak’s multiple comparison test for within-cell line comparison, or by Tukey’s multiple comparison test for between-cell line comparison. Results are expressed as mean ± SD, and each experiment has been performed at least in three biological replicates.

## 3 Results

### 3.1 NKG2DLs are differentially expressed in breast cancer and bone metastasis tissues

**FIGURE 1:**
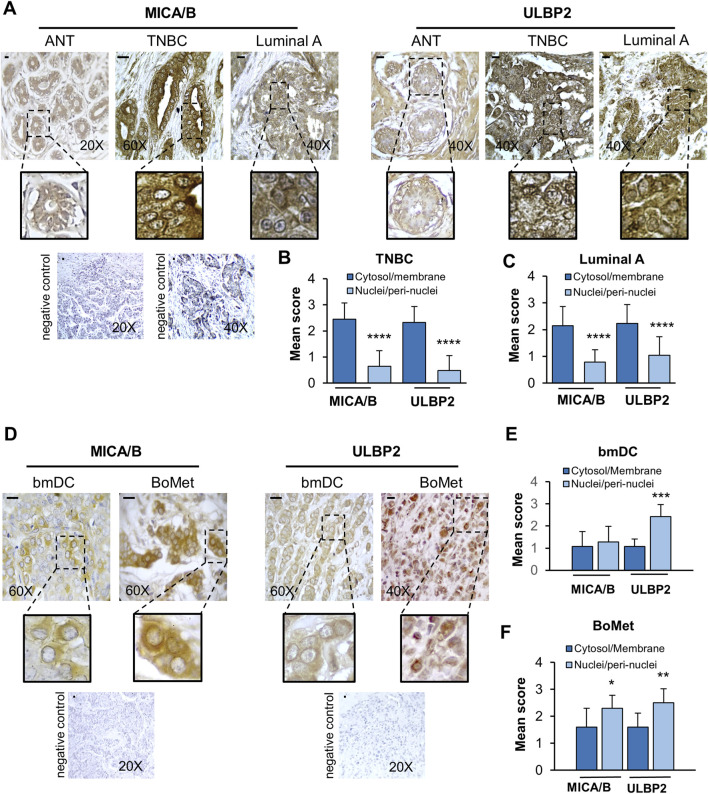
MICA/B and ULBP2 show different localizations according to the tumor stage. **(A)** Representative immunohistochemical staining of MICA/B and ULBP2 is shown in adjacent normal tissues (ANT; n = 20), triple-negative breast cancer (TNBC; n = 33) and Luminal A type breast cancer (n = 47). Scale bar = 10 μm. **(B,C)** Mean score of the distribution of NKG2SLs in the cytosol/membrane fraction compared to the nuclei/perinuclear fraction in either TNBC or Luminal A breast cancer. **(D)** Representative immunohistochemical staining of MICA/B and ULBP2 is shown in bone metastasis (BoMet; n = 10) and paired bone metastatic ductal carcinoma (bmDC; n = 7). Scale bar = 10 μm. **(E,F)** Mean score of the distribution of NKG2SLs in the cytosol/membrane fraction compared to the nuclei/perinuclear fraction. Mean of scores derived from the semi-quantitative analysis for MICA/B and ULBP2. Each bar represents the mean ± SD of the specimens, *p < 0.05, **p < 0.01, ***p < 0.001, and ****p < 0.0001.

As an initial approach in our study, we analyzed the expression of NKG2D ligands (MICA/B and ULBP2) in human tissues using IHC. Tissue samples included: adjacent normal breast tissue (ANT, n = 20), two subtypes of breast cancer—triple-negative breast cancer (TNBC, n = 33) and Luminal A (n = 47), as well as BoMet (n = 10) and bmDC samples (n = 7). The clinical and pathological characteristics of patients with bone metastases are reported in the Supplementary Table S1. Representative immunohistochemical images are presented in Figures 1A,D. The corresponding histograms display the mean scores from semi-quantitative staining analyzes and highlight significant statistical differences between cytosolic/membrane and nuclear/peri-nuclear distribution in each group (Figures 1B,C–E,F). In ANT, MICA/B and ULBP2 staining were primarily localized at the ductal level. In both TNBC and Luminal A samples, MICA/B and ULBP2 expression was predominantly localized to the cytoplasm and cell membrane with respect to the expression at the nuclear level (p < 0.0001). In BoMet tissues and their bmDC samples, the expression of NKG2D ligands was mainly localized to the perinuclear/nuclear region rather than the cytoplasm or membrane. This nuclear/perinuclear localization was statistically significant in BoMet for both MICA/B (p = 0.019) and ULBP2 (p = 0.005). In the matched primary BC tissues, statistical significance was observed only for ULBP2 (p = 0.0006).

Overall, our findings indicate a significant change in the localization of NKG2D ligands. In normal tissue and primary BC tissues (which showed no metastases at the time of biopsy), these ligands were primarily found in the cytosol and associated with the membranes. In contrast, in BoMet and in their bmDC, the NKG2D ligands were mostly located in the perinuclear/nuclear region. This pattern suggests a potential immune evasion strategy that aggressive breast cancers may employ to escape detection by NK and avoid immune surveillance.

### 3.2 NKG2DLs are expressed both on cell surface and intracellular in breast cancer cells

**FIGURE 2:**
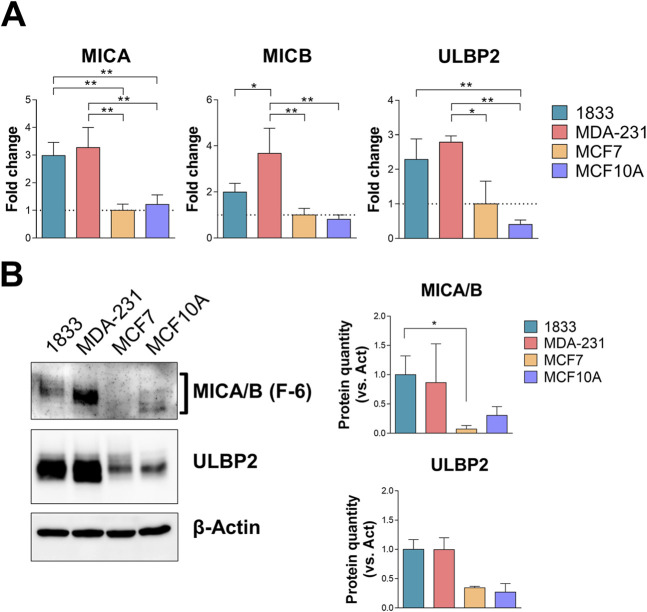
MICA/B and ULBP2 are differentially expressed in BoMet and BC cell lines with different invasive potential. **(A)** mRNA expression levels of the NKG2DLs in different BC cell lines (1833, MDA-231, and MCF7) and non-tumorigenic breast cell line (MCF10A). Results are expressed as 2^−ΔΔCt^ compared to MCF7. ONE-way ANOVA: *p < 0.05; **p < 0.01; n = 3. **(B)** Western blotting analysis and bands intensity quantification of MICA/B and ULBP2 in BC cell lysates. The expression of MICA/B and ULBP2 was normalized on β-actin. Statistical analysis was performed using Kruskall-Wallis test for non-parametric distribution: *p < 0.05, n = 3.

The expression levels of MICA/B and ULBP2 were then assessed in BC cell lines with different invasive potential (MDA-231, highly invasive, and MCF7, non-invasive), in a BoMet cell line (1833), and in normal breast cell line (MCF10A), cell lines that reflect the different human tissues analyzed before. MDA-231 cells expressed significantly higher levels of MICA, MICB and ULBP2 genes compared to non-invasive BC cells (MCF7) (p = 0.002 for MICA, p = 0.003 for MICB and p = 0.02 for ULBP2) or MCF10A (p = 0.003, p = 0.002, and p = 0.004, respectively), and only MICB gene compared to 1833 (p = 0.038) (Figure 2A). MICA and ULBP2 expression levels were significantly increased in 1833 compared to MCF7 (p = 0.004) and MCF10A (p = 0.009) or only MCF10A (p = 0.009), respectively. Although MCF10A cells showed lower levels of MICB and ULBP2 expression compared to MCF7, these differences were not statistically significant.

The increased gene expression levels of the NKG2DLs in most invasive and metastatic BC cells compared to non-invasive BC or normal breast cells is also reflected in the protein expression levels: 1833 and MDA-231 cells showed increased expression of the two NKG2DLs, even if the significance was reached only for MICA/B in 1833 versus MCF7 cells (p = 0.039) (Figure 2B).

Given the different expression of NKG2DLs in BC cells, which reflects the differences observed in tissues across BC subtypes, we further investigated the cellular distribution of MICA/B and ULBP2 in BC cells to identify further similarities with their tissue expression.

**FIGURE 3:**
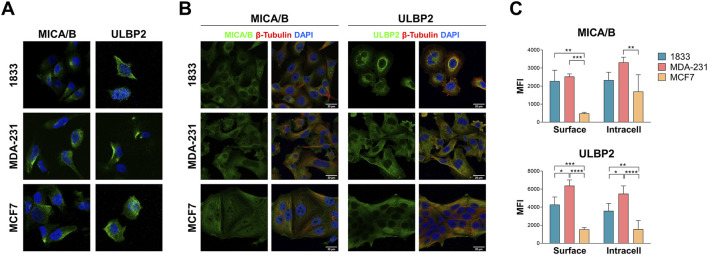
MICA/B and ULBP2 are expressed both on the surface and intracellularly in BoMet and BC cell lines with different invasive potential. **(A)** Surface staining of MICA/B and ULBP2 (green) in non-permeabilized breast cancer cells. Nuclei are stained with DAPI (blue). **(B)** Immunofluorescence detection of intracellular MICA/B and ULBP2 (green) in breast cancer cell lines. β-tubulin is marked in red; nuclei are stained with DAPI (blue). **(C)** Flow cytometry analysis of cell surface and intracellular MICA/B and ULBP2 expression. For surface staining cells were fixed with PFA and incubated with MICA/B-APC or ULBP2-PE primary antibody. For intracellular staining cells were fixed, permeabilized with 0.2% Tween-20, and incubated with primary antibody. Results are expressed as median fluorescence intensity (MFI) of each target after its isotype control subtraction. Intra-group statistical analysis was performed using ONE-way ANOVA with Tuckey’s multiple comparisons test: *p < 0.05; **p < 0.01; ***p < 0.001; ****p < 0.0001; n = 3.

MICA/B and ULBP2 are expressed on the cell surface of all cell lines (Figure 3A). The intracellular distribution showed that in MCF7 both MICA/B and ULBP2 are evenly distributed into the cytosol; in MDA-231 and in 1833 the two ligands are also distributed into the cytosol, but with a strong localization in the area around the nucleus, indicating a perinuclear distribution (Figure 3B). Additionally, intracellular ULBP2, and at a lesser extent MICA/B, seem to partly co-localize with β-tubulin.

NKG2DLs expression both on cell surface and intracellularly was also determined by flow cytometry: MICA/B showed a significant increase on cell surface in 1833 and MDA-231 compared to MCF7 (p = 0.022 and p = 0.024, respectively), while the same trend was observed intracellularly, although not significantly (Figure 3C). ULBP2, on the other side, showed almost two-fold higher expression both at the cell surface and intracellularly in 1833 and MDA-231 compared to MCF7 (p < 0.001 or p < 0.0001, as indicated in the figure).

These data suggest that MICA/B and ULBP2 are differentially expressed in BC cells with different invasive capacities and that these cell surface ligands are also expressed intracellularly.

### 3.3 NKG2DLs are retained in the Golgi apparatus in invasive breast cancer cells

**FIGURE 4:**
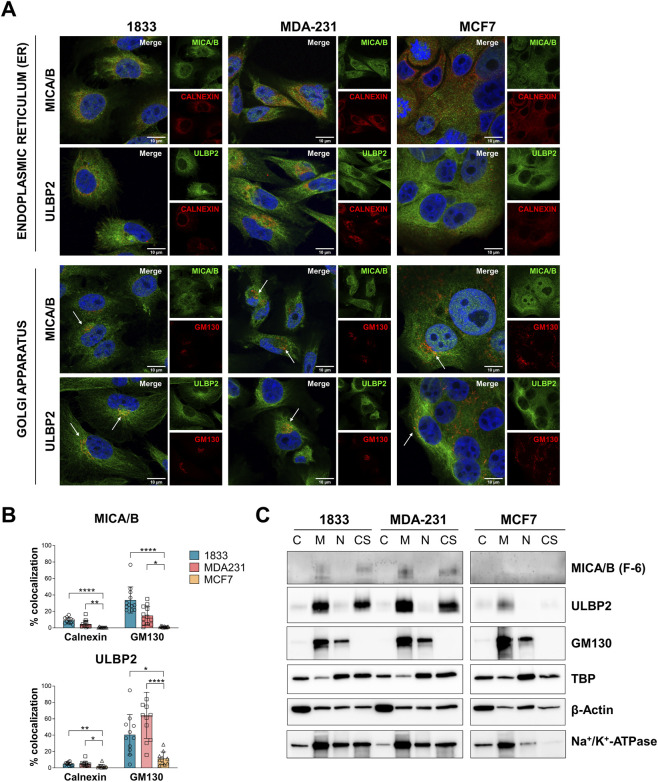
Intracellular MICA/B and ULBP2 co-localize with Golgi apparatus and show different mechanism of intracellular trafficking. **(A)** Subcellular localization of MICA/B and ULBP2 (green) in BC cell lines, and co-localization with calnexin (endoplasmic reticulum, ER) and GM130 (Golgi) (red). Nuclei are counterstained with DAPI (blue). White arrows indicate co-localization (yellow/orange staining). **(B)** Calnexin or GM130 co-localization with MICA/B or ULBP2 was quantified by ImageJ plug-in JACOP. Experiment was performed twice and analyzed in at least 6 fields for each condition. Statistical analysis was performed using Kruskall-Wallis for non-parametric distribution with Dunn’s multiple comparisons within organelles: *p < 0.05; **p < 0.01; ***p < 0.001; ****p < 0.0001. **(C)** Western blotting analysis of NKG2DLs and GM130 in subcellular fractions: cytosol (C), membrane (M), nucleus (N) and cytoskeleton (CS) extracts. TATA-binding protein (TBP), β-Actin and Na^+^/K^+^-ATPase were used as housekeeping for each fraction.

Next, we investigated more in detail the intracellular localization of these two proteins, which are typically expressed on cell surface. Considering their perinuclear localization, we then analyzed their co-localization with markers of the endoplasmic reticulum (ER) and Golgi apparatus, calnexin and GM130, respectively (Figure 4A). GM130 showed a greater co-localization with both MICA/B and ULBP2 compared to calnexin in all cell lines. In addition, a significant difference in the co-localization pattern was observed among the 3 cell lines. In particular, MICA/B and ULBP2 showed a significantly higher degree of co-localization with both GM130 and calnexin in 1833 and MDA-231, compared to MCF7 cells (from p < 0.05 to p < 0.0001, as indicated in the figure) (Figure 4B). In 1833, approximately 40% of GM130 co-localized with MICA/B and ULBP2, whereas in MDA-231, GM130 co-localized with 20% of MICA/B and 60% of ULBP2.

To further support these results, cell fractionation was performed to detect MICA/B and ULBP2 in the different sub-cellular extracts. In both 1833 and in MDA-231, MICA/B and ULBP2 were slightly enriched in the cytosolic fraction and more abundantly in the membrane and in the cytoskeletal fractions (Figure 4C). In contrast, MCF7 showed minimal expression of MICA/B, while ULBP2 was detected in the membrane fraction but at lower levels than the more aggressive cell lines. The membrane fraction includes both plasma membrane and membrane of the intracellular organelles, such as the Golgi apparatus, as confirmed by the presence of GM130, further supporting the retention of MICA/B and ULBP2 in the Golgi apparatus.

Overall, these results suggest that MICA/B and ULBP2 are primarily retained intracellularly, especially within the Golgi apparatus, in the more aggressive BC cells (MDA-231 and 1833). This retention within cells could be suggestive evidence of a mechanism useful for avoiding immune recognition. In contrast, the less aggressive MCF7 cells exhibit lower overall expression levels of MICA/B and ULBP2, along with reduced co-localization with ER and Golgi apparatus markers, indicating different intracellular processing and fate of NKG2D ligands in these cells.

Finally, the enrichment of these proteins in the cytoskeletal fraction also suggests their involvement in distinct trafficking and recycling pathways among the different cell types.

### 3.4 Analysis of NKG2DLs N-glycosylation by EndoH and PNGase F digestion in breast cancer cells lysates

**FIGURE 5:**
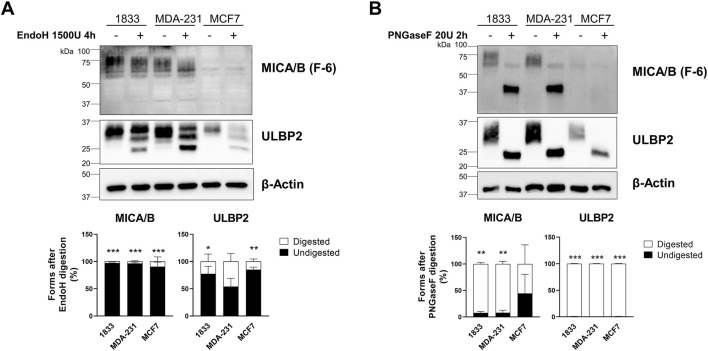
MICA/B and ULBP2 are resistant to EndoH digestion but sensitive to PNGase F digestion. **(A)** Western blotting analysis of MICA/B and ULBP2 in cell lysates from BC cell lines, untreated or treated with 1500 U EndoH for 4 h. The bars represent the mean percentage of digested (□) and undigested (■) MICA/B and ULBP2 normalized on β-actin. Statistical analysis was performed using two-way ANOVA followed by Sidak’s multiple comparison test within cell line: *p < 0.05; **p < 0.001; ***p < 0.0001; n = 3. **(B)** Western blotting analysis of MICA/B and ULBP2 in cell lysates from BC cell lines, untreated or treated with 20 U PNGase F for 2 h. The bars represent the mean percentage of digested (□) and undigested (■) MICA/B and ULBP2 normalized on β-actin. Statistical analysis was performed using two-way ANOVA followed by Sidak’s multiple comparison test within cell line: **p < 0.001; ***p < 0.0001; n = 3.

To determine whether retained NKG2DLs are immature or ER–Golgi arrested we assess ligand maturation and trafficking by de-glycosylating enzyme digestion. Cell lysates from 1833, MDA-231, and MCF7 breast cancer cells were digested with EndoH or PNGase F. EndoH treatment did not induce any detectable change in the electrophoretic mobility of MICA/B in any of the breast cancer cell lines analyzed, indicating complete resistance to the enzyme. In contrast, ULBP2 showed a very limited sensitivity to EndoH digestion, as indicated by the presence of a minor lower–molecular-weight band; however, this effect was not statistically significant (Figure 5A). In contrast to EndoH, PNGase F treatment resulted in a marked shift toward lower–molecular-weight forms of both ligands. ULBP2 was completely sensitive to PNGase F digestion in all cell lines, with a statistically significant increase in the digested form. MICA/B was almost completely digested in 1833 and MDA-231 cells showed statistically significant reduction in the undigested form. Conversely, MCF7 cells retained approximately 44.5% of undigested MICA/B following PNGase F treatment; however, this difference did not reach statistical significance (Figure 5B). Overall, these results indicate that MICA/B and ULBP2 mainly have complex N-linked glycans, even if the extent of PNGase F sensitivity varies between cell lines.

### 3.5 Metastatic breast cancer cells rely on efficient N-glycosilation for NKG2DLs engagement on cell surface

**FIGURE 6:**
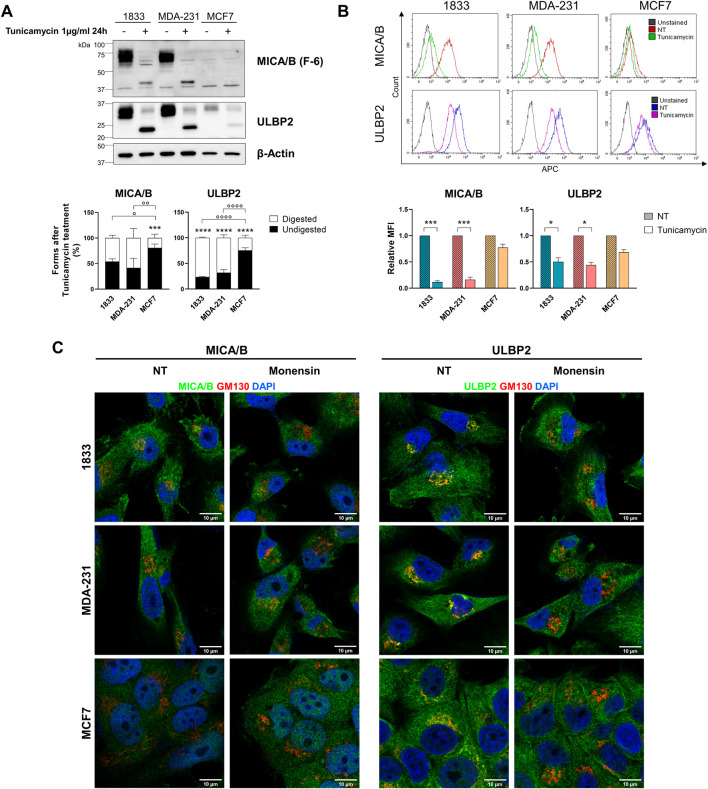
NKG2DLs glycosylation and surface localization are differentially affected by tunicamycin in metastatic and non-metastatic BC cells and depend on intact Golgi for intracellular retention. **(A)** Western blotting analysis and bands intensity quantification of MICA/B and ULBP2 in BC cells after treatment with 1500 U tunicamycin for 24 h. The bars represent the mean percentage of digested (□) and undigested (■) MICA/B and ULBP2 normalized on β-actin. Statistical analysis was performed using two-way ANOVA followed by Sidak’s multiple comparison test within cell line: ***p < 0.0001; or using two-way ANOVA followed by Tukey’s multiple comparison test between cell lines: °p < 0.05; °°p < 0.01;   °°°p < 0.001;    °°°°p < 0.0001; n = 3. **(B)** Flow cytometry analysis of cell surface MICA/B and ULBP2 expression after treatment with 1500 U tunicamycin for 24 h. For surface staining cells were incubated with MICA/B-APC or ULBP2-APC primary antibody. Representative histograms overlay are shown for each cell line. Results are expressed as relative median fluorescence intensity (MFI) of each target to its untreated control (NT). Statistical analysis was performed using ONE-way ANOVA with Sidak’s multiple comparisons test within cell lines: *p < 0.05; ***p < 0.001; n = 3. **(C)** Subcellular localization of MICA/B and ULBP2 (green) in BC cell lines, and co-localization with GM130 (Golgi) (red) after treatment with 2 µM monensin for 3 h. Nuclei are counterstained with DAPI (blue).

Tunicamycin, an inducer of endoplasmic reticulum stress, inhibits *de novo* N-glycosylation. Following treatment, we observed marked differences in tunicamycin sensitivity among the analysed breast cancer cell lines. Specifically, 1833 and MDA-231 cells exhibited significantly higher sensitivity to tunicamycin compared with MCF7 cells (Figure 6A). Additionally, treatment with tunicamycin significantly reduced surface expression of MICA/B in 1833 and MDA-231 (89% and 84% reduction, respectively), whereas only a modest decrease (23%) was observed in MCF7 cells (Figure 6B). Conversely, surface expression of ULBP2 was less affected, showing decreases of 48%, 56%, and 33% in 1833, MDA-231 and MCF7, respectively, though significantly only in invasive and metastatic BC cell lines. These findings suggest that ULBP2 seems to be less dependent on a complete and functional glycosylation status for efficient trafficking to the cell surface.

Additionally, treatment with monensin resulted in the loss of MICA/B and ULBP2 co-localization with the Golgi marker GM130 in all cell lines (Figure 6C), indicating that intracellular retention of NKG2DLs depends on the integrity of Golgi architecture.

## 4 Discussion

Our study describes differences in the intracellular localization of MICA/B and ULBP2 across breast cancer subtypes and corresponding cell lines, potentially reflecting variation in immune evasion strategies.

In bone metastatic ductal carcinoma, in bone metastasis and in the corresponding invasive cell lines (MDA-231 and 1833), MICA/B and ULBP2 showed a clear perinuclear localization, which contrasts with the primarily cytosolic distribution observed in non-metastatic (at the time of biopsy collection) BC tissues and the less aggressive MCF7 cell line. This perinuclear pattern was further supported by experiments of cell fraction, showing enrichment of NKG2DLs in the membrane and cytoskeleton-associated fractions in the metastatic lines. Importantly, co-localization with the Golgi apparatus could be suggestive evidence that these ligands may be retained intracellularly, rather than trafficked to the cell surface.

MICA/B and ULBP2 are expressed in the normal ducts of breast tissues. In samples from TNBC and Luminal A subtypes, which were non-metastatic at the time of biopsy collection, these proteins are primarily found in the cytosol and on the membrane. Notably, the perinuclear and nuclear localization observed in bmDC, along with their matched BoMet, suggests that this mechanism may facilitate the most aggressive cells in evading immune surveillance. The intracellular retention of MICA/B in BC tissues has also been reported by other researchers (Ghadially et al., 2017). Furthermore, MICB is strongly present intracellularly also in neuroblastoma (Raffaghello et al., 2004), as well as MICA/B in renal cell carcinoma (Sconocchia et al., 2009). In several human melanoma cells, low or absent expression of MICA on the cell surface has been observed along with the intracellular accumulation of immature forms of MICA in the endoplasmic reticulum. This mechanism is thought to protect the cells from NKG2D-mediated killing by natural killer (NK) cells (Fuertes et al., 2008).

NKG2D ligands expression is regulated at post-transcriptional, translational, and post-translational levels. It has been demonstrated that at post-translational level, ubiquitination, acylation and glycosylation may occur and these different modifications regulates the intracellular retention and surface expression and distribution (Wang et al., 2024). Indeed, S-acylation, specifically palmitoylation, of MICA is responsible for efficient recognition and cytolysis by human NK cells (Eleme et al., 2004), while another group demonstrated that palmitoylation of MICA is responsible for its recruitment at cholesterol-rich microdomains in the plasma membrane, and it is essential for efficient MICA shedding (Aguera-Gon et al., 2011). S-acylation occurs at a dicystein motif at position 331 and 332 in proximity of the transmembrane domain. However, ULBP2, which does not have a transmembrane domain but it is attached to the plasma membrane through a glycosylphosphatidylinositol (GPI)-anchor, is not acylated. MICA ubiquitination redistributes the ligand from the plasma membrane to an intracellular compartment but not leading to its degradation (Thomas et al., 2008). Another post-translational modification to which MICA/B and ULBPs are subjected is N-glycosylation. NKG2DLs require proper N-linked glycosylation to ensure accurate protein folding, maintain structural stability, and enable efficient trafficking through the secretory pathway (Andresen et al., 2012; Lin et al., 2024). This post-translational modification is critical for the maturation of the ligands and their subsequent delivery to the cell surface. Impairment of glycosylation processes may result in the retention of NKG2DLs within the Golgi apparatus, attributable to the activation of intracellular quality control mechanisms. In our experimental cell lines, MICA/B proteins exhibited variable molecular weights, with slower migration rate in MCF7 cells compared to MDA-231 and 1833 cells. This difference likely reflects variations in the extent of glycosylation among the cell lines. Additionally, MDA-231 and 1833 are the cell lines that showed higher degree of co-localization of MICA/B and ULBP2 with the Golgi apparatus, further suggesting an involvement and alteration of N-glycosylation in the processing and maturation of the NKG2D ligands in metastatic cells.

Since during their trafficking through the ER and Golgi apparatus, NKG2DLs undergo refolding or are subject to various post-translational modifications (PTMs), Golgi apparatus dysregulation, and consequently PTMs dysfunctions, can contribute to intracellular retention by impairing protein maturation (Schmiedel and Mandelboim, 2018). For example, it has been previously demonstrated that MICB and ULBP2 are retained in the ER and *cis*-Golgi in human cytomegalovirus (HCMV) infected fibroblasts and only MICA in melanoma cells. This intracellular retention prevents these protein from undergoing proper maturation, resulting in EndoH-sensitive forms (Fuertes et al., 2008; Dunn et al., 2003). This retention of immature forms results in the reduced expression of these proteins on the cell surface, increasing resistance to NK cell cytotoxicity.

In our study, Endo H and PNGase F treatments yielded consistent results across all breast cancer cell lines, with both NKG2DLs sensitive to PNGase F but resistant to Endo H, indicating that they are modified by complex N-linked glycans and that they efficiently exit from the ER. In contrast, tunicamycin treatment revealed differences between metastatic and non-metastatic cells: NKG2DLs were sensitive in metastatic lines (1833 and MDA-231) but largely resistant in non-metastatic MCF7 cells. These findings suggest that, although NKG2DLs progress beyond the ER in all cell lines, their intracellular handling may differ with metastatic phenotype, potentially involving differential regulation at the Golgi level and influencing NKG2D-mediated NK cell recognition.

The Golgi apparatus is essential in cell homeostasis for correct proteins and lipids modification and trafficking towards different cellular destinations. The orientation of the Golgi apparatus, along with vesicular trafficking and secretion, becomes dysregulated during malignant transformation. This dysregulation is a significant characteristic of cancer. An abnormal Golgi apparatus can affect the tumor microenvironment and the immune landscape, contributing to increased invasion and metastatic potential (Bajaj et al., 2022; Zhang, 2021). Although Golgi retention of NKG2DLs is not exclusive to breast cancer, it appears particularly relevant in breast cancer cells with bone metastatic potential (Liang et al., 2023). Within the bone microenvironment, this Golgi-dependent mechanism could further limit NK-cell activation and facilitate metastatic colonization. Thus, Golgi remodeling in breast cancer cells may suggest an adaptive response to the immune constraints of the bone niche. In the context of bone metastasis, such changes may impair NK-cell or other immune effector infiltration by reducing tumor-cell presentation of activating ligands (e.g., NKG2DLs) or by altering the tumor secretome to create an immune-excluded microenvironment. Thus, Golgi or glycosylation defects may represent a mechanism that links tumor-intrinsic secretory/trafficking dysfunction with changes in the metastatic microenvironment (Gupta et al., 2023).

**FIGURE 7:**
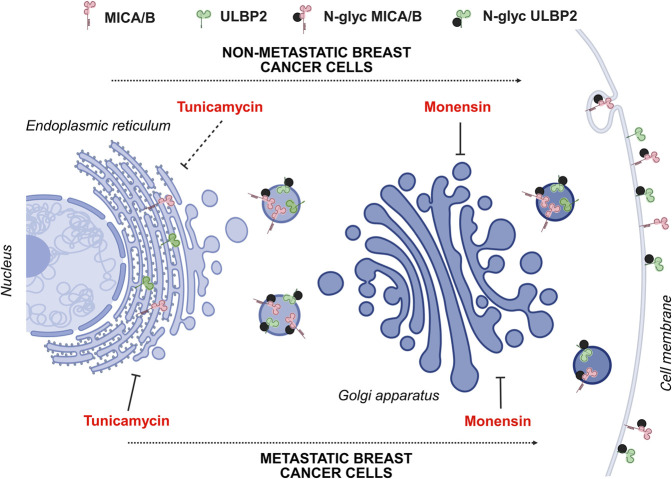
Schematic representation of MICA/B and ULBP2 maturation and trafficking through Golgi apparatus in metastatic and non-metastatic BC cell lines. In metastatic cell lines (1833 and MDA-231), following protein synthesis, NKG2DLs acquire complex N-glycans and traffic from the endoplasmic reticulum (ER) to the Golgi apparatus. When N-glycosylation is impaired (e.g., by tunicamycin treatment), NKG2DLs fail to reach the cell surface. In contrast, in the non-metastatic MCF7 cell line, which is largely resistant to tunicamycin, immature NKG2DLs are still transported through the Golgi apparatus and delivered to the plasma membrane, resulting in increased surface expression irrespective of correct glycosylation. Nevertheless, regardless of glycosylation status, delivery to the plasma membrane requires an intact Golgi architecture, as demonstrated by the loss of co-localization with a Golgi marker following monensin treatment. Created in BioRender. Lombardi, G. (2025) https://BioRender.com/jnrm8ui.

Secreted, transmembrane and membrane-associated protein needs to be delivered directly to the cell surface. In post-Golgi trafficking towards plasma membrane, as well as in retrograde trafficking from Golgi apparatus to ER, microtubules play a crucial role both in reorganizing the cytoskeleton and in vesicle transport (Stalder and Gershlick, 2020; Fucini et al., 2002). Our results showed that MICA/B and ULBP2 co-localize with the Golgi apparatus, rather than the ER, are enriched in the membrane fraction as well as in the cytoskeletal protein fraction, and their surface expression is reduced upon tunicamycin treatment in MDA-231 and 1833. These findings indicate that MICA/B and ULBP2 are retained in the Golgi apparatus where they undergo post-translational modification (i.e., N-glycosylation), and then only correctly glycosylated proteins are transported to the cell surface through microtubules, in invasive and metastatic BC cells. On the other side, non-metastatic MCF7 cells exhibit limited enrichment of MICA/B and ULBP2 in the cytoskeletal fraction, reduced co-localization with β-tubulin, as well as increased cell surface localization after tunicamycin treatment, suggesting differences in the mechanism of NKG2DLs processing, trafficking, and recycling when compared to more aggressive and invasive BC cells (Figure 7).

Our findings raise the possibility that NKG2DLs retention may influence the immune landscape of bone metastases. NKG2D–NKG2DL interactions are key determinants of NK cell recognition and activation; thus, altered retention or shedding of NKG2DLs could impact NK cell infiltration and effector function within the metastatic niche. Sustained ligand expression may enhance NK-mediated cytotoxicity and cytokine production, whereas excessive retention or dysregulated expression could contribute to immune evasion by desensitizing NK cells or altering their recruitment. Further studies assessing NK cell phenotype and spatial distribution in relation to NKG2DL expression in bone lesions would shed light on this important mechanistic insight.

Considering the different response to NKG2DLs glycosylation status between metastatic and non-metastatic BC cells, we can speculate that components of the glycosylation and trafficking machinery may represent indirect targets to modulate NKG2DL surface expression and enhance NK cell–mediated antitumor responses (Rodriguez, 2025; Yu, 2023; Ren et al., 2024). Although preliminary, these observations support further investigation into glycosylation-dependent regulation of NKG2DLs as a strategy to improve immunotherapeutic approaches in breast cancer metastasis.

A limitation of this study is the small number of bmDC cases paired with corresponding bone metastases. Access to bone metastatic tissue is inherently limited, as biopsies are typically obtained only during orthopaedic surgery for stabilization or pathological fractures, which applies to a restricted subset of patients. In addition, the often-long interval between primary breast cancer diagnosis and the development of bone metastases hampers the retrieval of matched primary tumours. Together, these factors reduce the number of analysable paired samples and may limit the statistical power and generalizability of the findings. While this study is primarily descriptive, and does not directly demonstrate impaired NK cell recognition or function, which remains central to the biological significance of NKG2D ligand retention, it establishes an important starting point for subsequent mechanistic and functional analyses. Despite these constraints, our study provides valuable insights into the biological characteristics of bone metastases derived from bmDC, as supported by *in vitro* experiments. Although based on a limited cohort, the study offers important evidence that may inform future research and clinical investigations in this field.

Further analyses are needed to dissect the trafficking of the NKG2DL from Golgi to the plasma membranes, and to define whether dysfunction in the NKG2DLs modification results in dysfunctional ligands that impair NK cell recognition or function. Overall, the intracellular retention of MICA/B and ULBP2 may serve as a powerful strategy employed by invasive and metastatic breast cancer cells, granting them a significant immunological advantage within the tumor microenvironment. This mechanism may enhance their ability to elude recognition and elimination by NK cells, ultimately supporting their survival and progression.

## Data Availability

The datasets presented in this study can be found in online repositories. The names of the repository/repositories and accession number(s) can be found below: https://doi.org/10.5281/zenodo.16407405.
