# Supplementary material for: Breast cancer bone metastasis and bone metastatic cells retain NKG2DLs intracellularly: could this be a strategy to evade immune recognition?
Source: Front Cell Dev Biol. 2026 Jan 27;14:1717607. doi: 10.3389/fcell.2026.1717607 (PMC12886486; doi:10.3389/fcell.2026.1717607)
Supplement: Supplementary file 1 [file Table1.docx]

***Supplementary Materials***

**1 SUPPLEMENTARY TABLE**

**1.1 Table S1** Clinical and pathological characteristics of patients with bone metastasis

| **Parameter** | **Cases** |
| --- | --- |
| **Age (years)** |  |
| < 50 | 1 |
| ≥ 50 | 9 |
| **ER** |  |
| negative | 1 |
| positive | 9 |
| **PR** |  |
| negative | 1 |
| positive | 9 |
| **HER2** |  |
| negative | 1 |
| positive | 9 |
| **Ki67** |  |
| <14 | 2 |
| >14 | 7 |
| n.d. | 1 |
| **Bone metastasis site** |  |
| vertebrae | 6 |
| femur | 2 |
| humerus | 1 |
| pelvis | 1 |
| **Molecular type** |  |
| Luminal A | 9 |
| TNBC | 1 |

n.d.: not determined
